# Spatiotemporal Analysis of Predation by Carabid Beetles (Carabidae) on Nematode Infected and Uninfected Slugs in the Field

**DOI:** 10.1371/journal.pone.0082142

**Published:** 2013-12-12

**Authors:** Bjørn Arild Hatteland, Solveig Haukeland, Steffen Roth, May Bente Brurberg, Ian P. Vaughan, William O. C. Symondson

**Affiliations:** 1 Department of Biology, University of Bergen, Bergen, Norway; 2 Horticulture and Urban Greening, Bioforsk - Norwegian Institute for Agricultural and Environmental Research, Ullensvang, Norway; 3 Plant Health and Plant Protection Division, Bioforsk - Norwegian Institute for Agricultural and Environmental Research, Ås, Norway; 4 University Museum of Bergen, University of Bergen, Bergen, Norway; 5 Cardiff School of Biosciences, Cardiff University, Cardiff, United Kingdom; University of California, Berkeley, United States of America

## Abstract

The dynamics of predation on parasites within prey has received relatively little attention despite the profound effects this is likely to have on both prey and parasite numbers and hence on biological control programmes where parasites are employed. The nematode *Phasmarhabditis hermaphrodita* is a commercially available biological agent against slugs. Predation on these slugs may, at the same time, result in intraguild predation on slug-parasitic nematodes. This study describes, for the first time, predation by carabid beetles on slugs and their nematode parasites on both spatial and temporal scales, using PCR-based methods. The highest nematode infection levels were found in the slugs *Deroceras reticulatum* and *Arion silvaticus*. Numbers of infected slugs decreased over time and no infected slugs were found four months after nematode application. The density of the most abundant slug, the invasive *Arion vulgaris*, was positively related to the activity-density of the carabid beetle, *Carabus nemoralis*. Predation on slugs was density and size related, with highest predation levels also on *A. vulgaris*. Predation on *A. vulgaris* decreased significantly in summer when these slugs were larger than one gram. Predation by *C. nemoralis* on slugs was opportunistic, without any preferences for specific species. Intraguild predation on the nematodes was low, suggesting that carabid beetles such as *C. nemoralis* probably do not have a significant impact on the success of biological control using *P. hermaphrodita*.

## Introduction

Trophic interactions are fundamental to ecology and spatial, as well as temporal, dynamics are essential to any understanding of such interactions [1]. Analyses of predator responses to prey densities, including pest species, are important when quantifying the role of predators in regulating pests. Most carabid beetles are polyphagous predators and their importance as natural enemies in agricultural ecosystems, as well as other ecosystems, has been studied extensively [2-4]. 

The carabid beetle *Pterostichus melanarius* Müller has been found to aggregate to areas with high slug densities and this phenomenon has been suggested as a direct and dynamic relationship rather than opportunistic predation [5]. In general, slugs are a major part of the diet in these beetles [6-8], and Symondson et al. [9] found a significant temporal effect of *P. melanarius* on slug population growth and a reverse effect on the nutritional status of beetles, and hence on their reproductive success. Most of these studies have focused only on the generalist *P. melanarius* which is an abundant species in arable fields. However, other carabids are also common in anthropogenic habitats (e.g. pastures, gardens), such as the relatively large species of the genus *Carabus*, and more importantly some of these are considered to be gastropod specialists [10-13]. Slug predation by large carabid species has become of increasing interest due to the need to identify potential natural enemies of the invasive slug *Arion vulgaris* [14]. *Arion vulgaris* Moquin-Tandon 1855 (also regarded as non-topotype *A. lusitanicus* Mabille 1868) has spread to many parts of northern Europe during the last decades [15,16] and is one of the most damaging gastropod pests in gardens, pastures and field crops [17,18].

Here we mainly focused on *Carabus nemoralis* Müller, a common carabid beetle in habitats where *A. vulgaris* is known to cause serious damage [19]. Previous work showed that *C. nemoralis* prefers small slugs as prey [20] and is highly active in spring when *A. vulgaris* is present as juveniles [21]. The spatial distribution and home range of *A. vulgaris* have been found to differ with density and age of the slugs; juveniles are more aggregated than adults and with higher densities leading to smaller home ranges [22]. Bohan et al. [5] showed non-opportunistic predation of several slug species by a carabid species leading to significant spatial correlation between prey and predator. Furthermore, a semi-field experiment testing the effect of releasing carabid beetles in plots with *A. vulgaris* showed that these beetles may cause a significant reduction in slug densities [23]. Based on these previous findings we hypothesize that predation by *C. nemoralis* on *A. vulgaris* is significant in spring when most of the slugs are in the juvenile phase < 1 g. Further, we wanted to test the hypothesis that *C. nemoralis* does not have a preference for any particular slug species as long as the slugs are abundant and < 1g. We also wanted to test whether the significant interaction patterns between *P. melanarius* and slugs, as found by Bohan et al. [5] and Bell et al. [24], also hold for other carabid-slug systems and whether these relationships change over time. We predicted aggregation of predators like *C. nemoralis* to higher densities of juvenile *A. vulgaris*.

The rhabditid nematode *Phasmarhabditis hermaphrodita* is a facultative parasite of terrestrial slugs and acts as a vector that transports associated lethal bacteria into the mantle of slugs [25–27]. The nematode is mass produced with the bacteria *Moraxella osloensis* and sold in many countries as an inundative biological control agent, primarily for use by gardeners but also high-value commercial crops [28,29]. *Phasmarhabditis hermaphrodita* are applied as dauer larvae to the soil surface and susceptible slug hosts are killed within 4 - 21 days depending on nematode numbers and temperature [30,31]. The nematode feeds and reproduces on the decaying slug, and on depletion of this food supply a new generation of dauer larvae are formed that actively disperse into the surrounding soil in search of new hosts. Our knowledge of the life-cycle is largely derived from laboratory studies on the host slug *D. reticulatum* [31,32]. Very little is known about the ecology of this nematode apart from a number of studies concerning field efficacy as a biocontrol agent and dispersal studies [30,32,33]. The behaviour of *P. hermaphrodita* and its persistence in the soil have until recently been difficult to study due to a lack of reliable detection and quantification assays. In the present paper we tested a recently developed method based on real-time PCR [34] to examine the persistence of *P. hermaphrodita* in field soil over time.

Intraguild predation occurs when two or more species that share the same host or prey attack each other [35]. Such predation may be unidirectional, should the beetles have a preference for slugs parasitized with nematodes. Predators may prefer to feed on debilitated parasitized prey due to increased handling efficiencies [36]. *Phasmarhabditis hermaphrodita* have already been shown to be prey of micro-arthropods such as Collembola and mites [37]. Carabids feeding on infected slugs may, by breaking transmission cycles, affect the success of nematodes as biological control agents. *Pterostichus melanarius* has been found to prefer nematode-infected over uninfected slugs in laboratory experiments when the slugs are alive, but avoided infected slug carcasses [38,39]. In the present work we analyse for the first time this tri-trophic interaction between carabids, nematodes and slugs directly in the field. 

The application of molecular methods to track trophic interactions is now widespread [6,40,41]. However, most of the studies in agricultural systems have been related to predation on herbivores, including pest species, while few have focused on intraguild predation [39,42,43]. Here we test the hypothesis that intraguild predation by carabids on nematode-infected slugs is significant in the field. At the same time, we are also using molecular methods to analyse predation rates on slugs on both a spatial and a temporal scale to test for species and size preferences.

## Material and Methods

All necessary permits were obtained for the described field study. The field was situated on a private owned land and permits were given by the land owner Ingemann Bernsen.

### Sampling scheme

**Figure 1:**
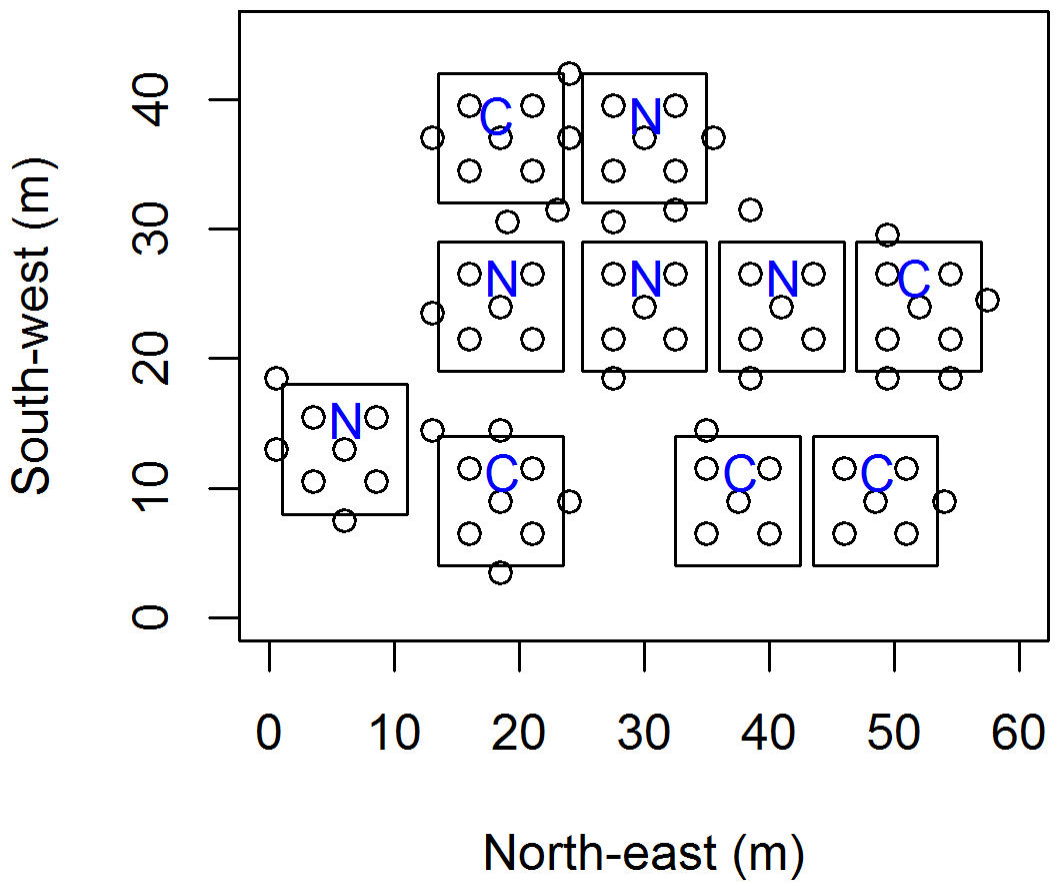
The spatial sample point pattern of the field used for gastropods and beetles. Each sample point is represented by a circle given by x and y coordinates based on distance (in meters). Treatment = nematode treatment.

Beetles and slugs were sampled in an abandoned meadow in a rural area outside Bergen, (60° 38’ N, 5°34’ E) close to small patches of deciduous trees. The meadow is mowed once a year. Molluscs, nematodes and beetles were sampled four times, 2-3 weeks apart. Dominant plants were the grasses *Deschampsia cespitosa* (L.), *Holcus lanatus* L., *Cardamine pratensis* L., *Rumex acetosa* L., *Alchemilla vulgaris* L. coll., *Epilobium angustifolium* L. and the moss species *Rhytidadelphus squarrosus* (Hedw.). In total, 75 sampling points were established within an area 54 x 43 m. Slugs were sampled at these points, each of which was associated with five pitfall traps for beetles. Fifty of these sampling points were located within 10 plots, each measuring 10 x 10 m and each containing five sampling points (Figure 1). The rest of the sampling points (25) were located randomly outside the plots but always at a distance of 1 m or more from a plot. This was mainly to check whether any infected slugs were found outside the nematode-treated squares, but also to increase the total number of samples. The first sampling of beetles was done in late April 2008 and was immediately followed by treatment with the slug-parasitic nematode *P. hermaphrodita* (26 April). The nematodes (available commercially as Nemaslug^®^) were applied to half (five) of the plots at a rate of 300 000 dauer larvae per m^2^ (Figure 1). The remaining plots were untreated controls. Repeated sampling of beetles post-treatment was carried out in early and late May as well as mid-June, yielding four sampling events in total. 

The beetles caught in the five pitfall traps at each sampling point during the 3-5 days of sampling period were pooled. They were sampled for three to five consecutive days before sampling molluscs. The different number of days of sampling was to achieve approximately the same number of *C. nemoralis* for each sampling date. The traps were checked each morning since these beetles are nocturnal. Beetles were transported back to the laboratory in plastic boxes with *c.* 2 cm of sphagnum moss peat to reduce regurgitations due to stress and killed at -20 °C. They were then transferred into tubes and stored at -80 °C. Only the known slug predators *C. nemoralis*, *P. melanarius* and *Pterostichus niger* were sampled. 

Molluscs were sampled by searching the vegetation down to the soil surface within 50 x 50 cm quadrats. Each quadrat was situated 50 cm from a set of five pitfall traps. All molluscs were counted and weighed to the nearest 0.1 g, except slugs < 0.1 g which were denoted as 0.05 g. Snails and slugs were released after weighing to avoid affecting population densities. Each repeated sampling event was always located within 50 cm of the pitfall traps, but using a new undisturbed piece of ground. In addition, at least 10 slugs were removed from each plot on each sample date and taken to the laboratory for dissection and detection of nematodes. 

Soil sampling was conducted 0, 1, 2, 4, 6, 8 and 18 weeks after the P. *hermaphrodita* treatment to give one sample per plot at each time period covering both treated plots and control plots. Each sample consisted of approximately 24 pooled cores of 2.5 cm dia., taken to a depth of approximately 15 cm. Cores were taken as evenly as possible in a ’W’ pattern within each plot. Nematodes were extracted from 200 gram soil samples using the elutriation technique [44], within a week after each sampling. Extracted nematodes were quantified using real-time PCR as described below. Soil moisture was also calculated at these sampling times.

### Feeding experiments for testing detectability of nematode and slug-DNA

To determine the efficiency of DNA detection of both the parasite and the host over time within the predator during digestion, we conducted a feeding experiment in which we fed *C. nemoralis* with *A. vulgaris* infected with *P. hermaphrodita* (see File S1). We also carried out two additional feeding experiments, also using *C. nemoralis*, feeding either on uninfected *A. distinctus* or *A. silvaticus*. The DNA detection period of the other target slugs (uninfected), *A. vulgaris* and *D. reticulatum*, have been tested previously using the same multiplex PCRs as applied in the present study [21].

### Beetle and slug dissections

The beetle foreguts were removed by forceps and weighed. The forceps were sterilized between dissections by cleaning it in 96% ethanol and open flame. The samples were stored at -80 °C prior to DNA extraction. 

The level of nematode infection was measured by dissecting the slugs by first washing in 70% ethanol, thereafter in sterile water, to kill and discard any nematodes on the surface of the slug. Dissections were made in physiological saline solution (0.25 strength Ringers solution, Merck) using forceps and a scalpel. Nematodes were observed by gently teasing the slug tissues and picking out nematodes under a binocular microscope (Leica MZ75). Nematodes were fixed in a triethanolamine formalin mixture (TAF) [28] for further morphological identification.

### Molecular analyses

We used two diagnostic multiplex PCR tests and one singleplex PCR test for detection of the different slug species within the guts of the carabids, as well as one standard and one real-time qPCR test for detection of the nematode *P. hermaphrodita* in beetles and soils respectively (Table 1, File S2). 

**Table 1:** Primers and probes for detection of slugs and nematodes.

**Species**	**Primer/probe name**	**Primer / Probe sequence**	**Fragment size (bp)**	**Annealing temperature (°C)**	**Reference**
*A. vulgaris*	A.l.-Co1-F1 (COI) A.l.-Co1-R2 (COI)	5´-GCCCCCATCTTTACTTTTACTTATTTGCTCC-3´ 5´-GCATAACCGCCCCCGATAATGGTATG-3´	310	51	Hatteland et al. 2011
*A. ater*	A.a.-Co1-F-new (COI)	5´-CACCACTGAGAGGAGCC-3´	225	51	Hatteland et al. 2011
*A. rufus*	A.r.-Co1-F1 (COI)	5´-MTTACTTATCGGTGCGC-3´	362	51	Hatteland et al. 2011
*A. ater* and *A. rufus*	A.a.-Co1-R1 (COI)	5´-GAAATGGACATAACCGACCTCG-3´			Hatteland et al. 2011
*A. silvaticus*	BH_1(COI) BH_2 (COI)	5´-TTTTGACTTCTACCACCTTCTCTT-3´ 5´-CGCCACTACGCCACTCA-3´	108	54	New
*Arion* spp.	Ai1F (12S)	5´-CACATAAATGATAGTCACC-3´	221	53	Dodd 2004
	AR2R (12S)	5´-TTTCTACCTGAACATTCATA-3´			
*D. reticulatum*	DR11F (12S)	5´-CTATACACAATTTTTAAATAAGC-3´	109	53	Dodd 2004
	DRF29RC (12S)	5´TGGTTATTATCTATTTGGTCTCTG-3´			
*P. hermaphrodita*	Ph-F-1754 (COI)	5´-TGGGTGCCCCTGATATAAGAT-3´	217	62	Read 2007
	Ph-R-(479-501, COI)	5´-GGCCAAATTCCAGATAAAACCCA-3´			
*P. hermaphrodita*	Ph 18S F (18S) Ph 18S R (18S) Ph probe (18S)	5’-CGGGCGTAGTTTGTTGACT-3’ 5’-ACAACCATGATAGGCCAATAGA-3’ 5’-FAM-TTCATCCGCTGAAGTCCGGAATTTT-TAMRA-3’	116	60.5	MacMillan et al. 2006

Gene amplified is given in brackets.

### Statistical analyses

Statistical analyses were performed in R (version 2.8.0) [45]. Generalized linear models (GLMs) were used to analyze the data from the controlled feeding experiments. As the data consisted of PCR-negatives and PCR-positives, a binomial distribution was used in the models. Median detection times (the time at which 50% of beetles tested positive [46], equivalent to the detectability half-life of [47] were calculated from the binomial regression equations.

Prey choice in the field was analysed by comparing slug-positive beetles with the densities of the various slug species using the Monte Carlo simulation model developed by Agusti et al. [48]. The null hypothesis was that slug consumption would be directly proportional to the densities of the respective slug species. Consumption events were thus randomly assigned to each beetle in turn with the relative probabilities of the different slug species being proportional to their field densities. The randomised frequency of consumption was calculated based on a negative binomial distribution with 20 000 iterations of the model, and predicted the number of beetles expected to test positive for each prey species. These values were compared with the observed consumption ratios of which any observed values falling outside the 95% confidence limits of the simulated numbers would indicate prey preferences. Analyses were carried out for April and early May separately, while the data from late May and June were combined due to low data sets. 

The spatial statistics were performed following Bivand et al. [49] and Baddeley [50]. Local coordinates of the field (x, y) were created by using the measured distance (in meters) between the sampling points and putting these measurements into a grid (Figure 1). Further, a spatial point data set was constructed by adding the coordinates to the sampled data. Spatial distribution of species was determined by Spatial Analysis by Distance IndicEs (SADIE), using the same coordinates with associated abundance of slugs or beetles in the free software SADIEShell (version 1.22). SADIE tests the departures from randomness resulting in an index of aggregation for a particular dataset. This index is based on an algorithm which incorporates a biological model to find the shortest distance to regularity by simulating the observed counts and comparing the permutated distances to regularity with the observed distances to regularity [51]. We used 153 randomization simulations with a standard random seed for all analyses. Spatial autocorrelation was found to be significantly negatively associated with distance (p < 0.001) using the Mantel test on distance/similarity matrices. 

Finally, generalised linear mixed-effect models (GLMMs) were used to test if beetle foregut mass (representing feeding history) was affected by the density of the various slug species, nematode variables and the number of slug-positive beetles of the various slugs. In addition, activity-density of beetles, nematode treatment and number of nematodes in the soil as well as proportion of infected slugs were used as explanatory variables for the density of slugs. The samples correlated with the spatial structure (x and y coordinates) were included as a random effect in the mixed-effect model to adjust for spatial dependence (autocorrelation) between samples. This was done by using the functions “corSpatial” and “glmmPQL” available in the packages “nlme” and “MASS” in R, respectively. The so-called penalized quasi-likelihood (PQL) allow for fitting the variance-covariance-matrix to the data, thus resulting in a spatial GLMM. These models were constructed according to the review by Dormann et al. [52] dealing with methods to account for spatial autocorrelation. GLMM approximation for analysing non-normal data such as counts has also recently been reviewed by Bolker et al. [53]. The quasipoisson distribution was used due to over-dispersion in the count data as suggested by diagnostic plots (leverage, normal Q-Q, fitted and scale location) and comparing the residual deviance with degrees of freedom. 

## Results

### Primer sensitivity and detectability of nematode and slug-DNA

**Figure 2:**
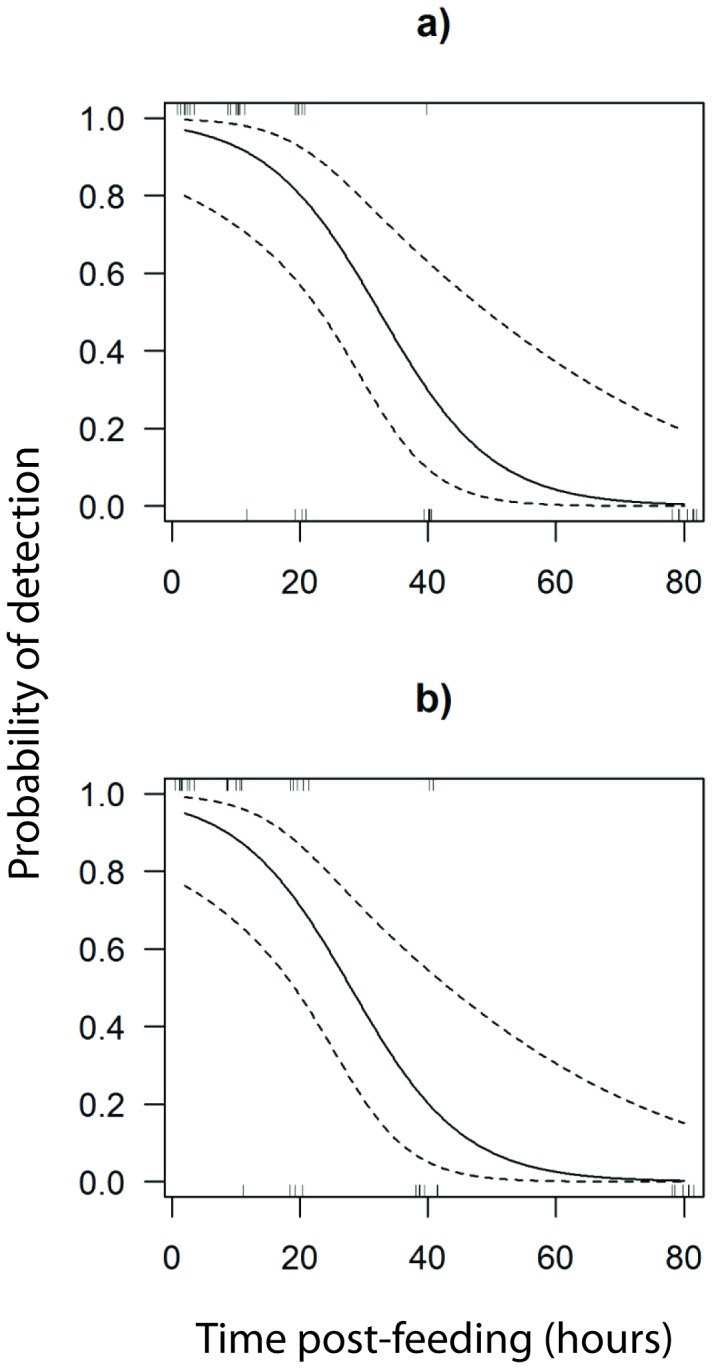
Detection period of prey DNA in the foreguts of *Carabus nemoralis* fed with *Arion* *distinctus* and *A. silvaticus* using the *12S rRNA* multiplex PCR and the singleplex PCR for *cox1*, respectively. The solid line represents the binomial model, while the dotted lines represent the upper and lower 95% confidence limits. The vertical lines represent the replicates and one line may in some cases consist of more than one replicate. (a) The detection of *A. distinctus* (*f* (E[y]) = 3.663 – 0.113x, AIC = 28.531, df = 37, p = 0.002, median detection period = 27.9 h). (b) The detection of *A. silvaticus* (*f* (E[y]) = 3.166 – 0.114x, AIC = 38.856, df = 37, p = 0.001, median detection period = 32.4 h).

None of the non-target organisms were co-amplified using the primer-pairs for *P. hermaphrodita* nematodes and *A. silvaticus* slugs, indicating that the primers are species-specific, at least in our field of potential prey and predators. In the feeding experiment *A. vulgaris* and *P. hermaphrodita* DNA were detected up to 24 h and 12 h, respectively, in foreguts of *C. nemoralis*. The juvenile slugs used in the feeding experiment were only moderately infected. After one week only 38% of the slugs (N=33) were actually infected with *P. hermaphrodita*, and the ones infected contained only a few nematodes (5.1±1.4 s.e.) as detected by dissection. The slugs *A. silvaticus* and *A. distinctus* were detected in the guts of beetles for up to 40h with median detection times of 27.9 h and 32.4 h, respectively (Figure 2).

### Densities in the field of carabid beetles and molluscs

In total, 201 adult carabid beetles were trapped, of which 174, 18 and 9 were *C. nemoralis*, *P. melanarius* and *P. niger*, respectively. The two *Pterostichus* species were almost exclusively caught in late May and June. All three species included individuals that were positive for slug DNA (Table 2). Due to the low numbers of the *Pterostichus* species, only *C. nemoralis* were included in the statistical analyses. Females and males of *C. nemoralis* were caught in similar numbers (female to male ratio approximately 0.9); males were more active in early May while females were more active in June. Foregut mass of females and males were in total 26±1.7 (s.e.) and 24±1.7 mg (all months pooled), respectively. *Carabus nemoralis* showed no significant aggregation across the site in any sampling period (Table 3), although a tendency of aggregation was found in June (*I*_*a*_ = 1.270, *P*_*a*_ = 0.093). 

**Table 2:** Carabid beetle predation on slugs as measured by number of beetles testing positive for slug DNA.

	***Carabus nemoralis*** (N=174)	***Pterostichus niger*** (N=9)	***Pterostichus melanarius*** (N=18)
***A. lusitanicus***	23	2	0
***A. distinctus***	5	0	0
***A. silvaticus***	16	0	0
***D. reticulatum***	13	1	1

**Table 3:** Results from the Spatial Analysis by Distance IndicEs (SADIE) of *Carabus nemoralis* and *Arion* *vulgaris*.

	**Index**	***Carabus nemoralis***	***Arion vulgaris***
**April**	*Ia*	1.026	1.238
	*Pa*	0.366	0.119
**Early May**	*Ia*	0.993	0.813
	*Pa*	0.445	0.881
**Late May**	*Ia*	0.870	1.097
	*Pa*	0.740	0.261
**June**	*Ia*	1.270	1.078
	*Pa*	0.093	0.289

*Ia* = Index of aggregation, *Pa* = Probability of aggregation.

In total, 3015 molluscs were collected, of which 896, 968, 229 and 473 were *Arion silvaticus*, *A. vulgaris*, *A. distinctus* and *D. reticulatum* respectively. All slugs were exclusively sampled from the quadrats and none were found in the pitfall traps together with carabid beetles. These species were the most common molluscs in the field and were used for statistical analyses. The number of all the four common slugs decreased considerably from late April to early May, while stabilizing in May except *A. silvaticus* that was still decreasing. The density of these slug species increased again in June (Table 4). *Arion vulgaris* was mainly in the juvenile phase in April and early May, hence possibly more prone to predation by beetles than later in the season. Like the beetles, the four most common slugs showed a random spatial distribution (Table 3).

**Table 4:** Predation in the field by *Carabus nemoralis* on the slugs *Arion* *vulgaris*, *A. distinctus*, *A. silvaticus* and *Deroceras reticulatum*, as well as intraguild predation on nematode-infected slugs using the nematode *Phasmarhabditis hermaphrodita*.

**No. beetles tested**	**Species Slug/nematode**	**PCR-positive *C. nemoralis* (%)**	**No. of slugs per m^2^**	**Mass of individual slugs (g)**
**April**	*A. vulgaris*	23.3	16.4±2.0	0.34±0.03
N=43	*A. distinctus*	9.3	5.3±2.4	0.23±0.01
	*A. silvaticus*	11.6	19.4±3.1	0.16±0.01
	*D. reticulatum*	7.0	10.8±2.4	0.28±0.01
**Early May**	*A. vulgaris*	20.0	10.0±1.2	0.66±0.07
N=50	*A. distinctus*	2.0	2.4±0.4	0.20±0.02
	*A. silvaticus*	4.0	9.9±1.3	0.14±0.01
	*D. reticulatum*	8.0	6.8±0.8	0.34±0.01
	*P. hermaphrodita*	4.0	-	-
**Late May**	*A. vulgaris*	6.8	10.0±1.2	1.26±0.12
N=44	*A. distinctus*	0	1.6±0.4	0.17±0.03
	*A. silvaticus*	6.8	5.7±1.0	0.17±0.01
	*D. reticulatum*	9.1	4.0±0.8	0.35±0.02
	*P. hermaphrodita*	0	-	-
**June**	*A. vulgaris*	2.9	11.6±1.2	2.19±0.30
N=36	*A. distinctus*	0	3.6±0.8	0.19±0.01
	*A. silvaticus*	14.3	16.9±1.9	0.17±0.01
	*D. reticulatum*	2.9	4.0±0.4	0.39±0.02
	*P. hermaphrodita*	2.7	-	-

Predation is given as PCR positive beetles for slug and nematode DNA of the respective species. Means ± standard error.

### Nematode-infected slugs and nematodes in the soil

A total of 486 slugs were dissected (*D. reticulatum A. vulgaris*, *A. silvaticus* and *A. distinctus*) comprising 257 from nematode treated plots and 229 from untreated control plots. *Phasmarhabditis hermaphrodita* infected slugs were only found in slugs from the nematode treated plots and numbers of infected slugs varied considerably between species. *Deroceras reticulatum* was most frequently infected while *Arion* species were less infected (Table 5). Only 40% of all dissected *A. vulgaris* were infected 12 days after nematode application and thereafter none. All dissected *D. reticulatum* were found to be infected at 12 days. The non-pest and native species *A. silvaticus* was also frequently infected by nematodes (50% at 12 days). We found similar proportions of infected slugs for both *A. silvaticus* and *D. reticulatum* (36-62%) about 1 and 2 months after nematode application (Table 5). *Arion distinctus* was present in low numbers in the samples, but were also found to be infected with *P. hermaphrodita* on two sampling dates 12 and 55 days after treatment (Table 5). For all slugs sampled after 4 months, none were infected with *P. hermaphrodita*. We observed only a few nematodes (typically 1-4) in all live slugs, while dead slugs had higher numbers of nematodes (> 10). The latter was typically found for *D. reticulatum*, where over half of the dissected slugs were dead with high numbers of nematodes after the first sampling event. On the other hand, most of the dissected *Arion* slugs were still alive and those that were infected had only low numbers of nematodes. 

**Table 5:** Phasmarhabditis hermaphrodita infection of four slug species: *Deroceras reticulatum*, *Arion* *distinctus*, *A. silvaticus* and *A. vulgaris*.

Slug species	Days after nematode application	% infected slugs	Mean number nematodes per slug	N	Comments
*D. reticulatum*	12	100	75.5±12.7	11	8 dead with nematode reproduction
*A. distinctus*	12	28	28.6±18.4	7	2 dead with nematode reproduction
*A. silvaticus*	12	50	1.9±0.6	14	All alive
*A. vulgaris*	12	40	14.3±9	15	2 dead with nematode reproduction
*D. reticulatum*	34	62	4.4±1.7	8	All alive
*A. distinctus*	34	0	0	2	All alive
*A. silvaticus*	34	39	2.6±0.9	18	All alive
*A. vulgaris*	34	0	0	21	All alive
*D. reticulatum*	55	36	1.8±0.7	22	All alive
*A. distinctus*	55	25	1.6±1.6	4	All alive
*A. silvaticus*	55	39	1.7±0.4	44	All alive
*A. vulgaris*	55	0	0	40	All alive
*D. reticulatum*	122	0	0	22	All alive
*A. distinctus*	122	0	0	2	All alive
*A. silvaticus*	122	0	0	11	All alive
*A. vulgaris*	122	0	0	16	All alive

Numbers are based on a total of 257 dissected slugs from 5 nematode-treated plots (10x10m). Means ± s.e. “N” = number of dissected slugs.

**Figure 3:**
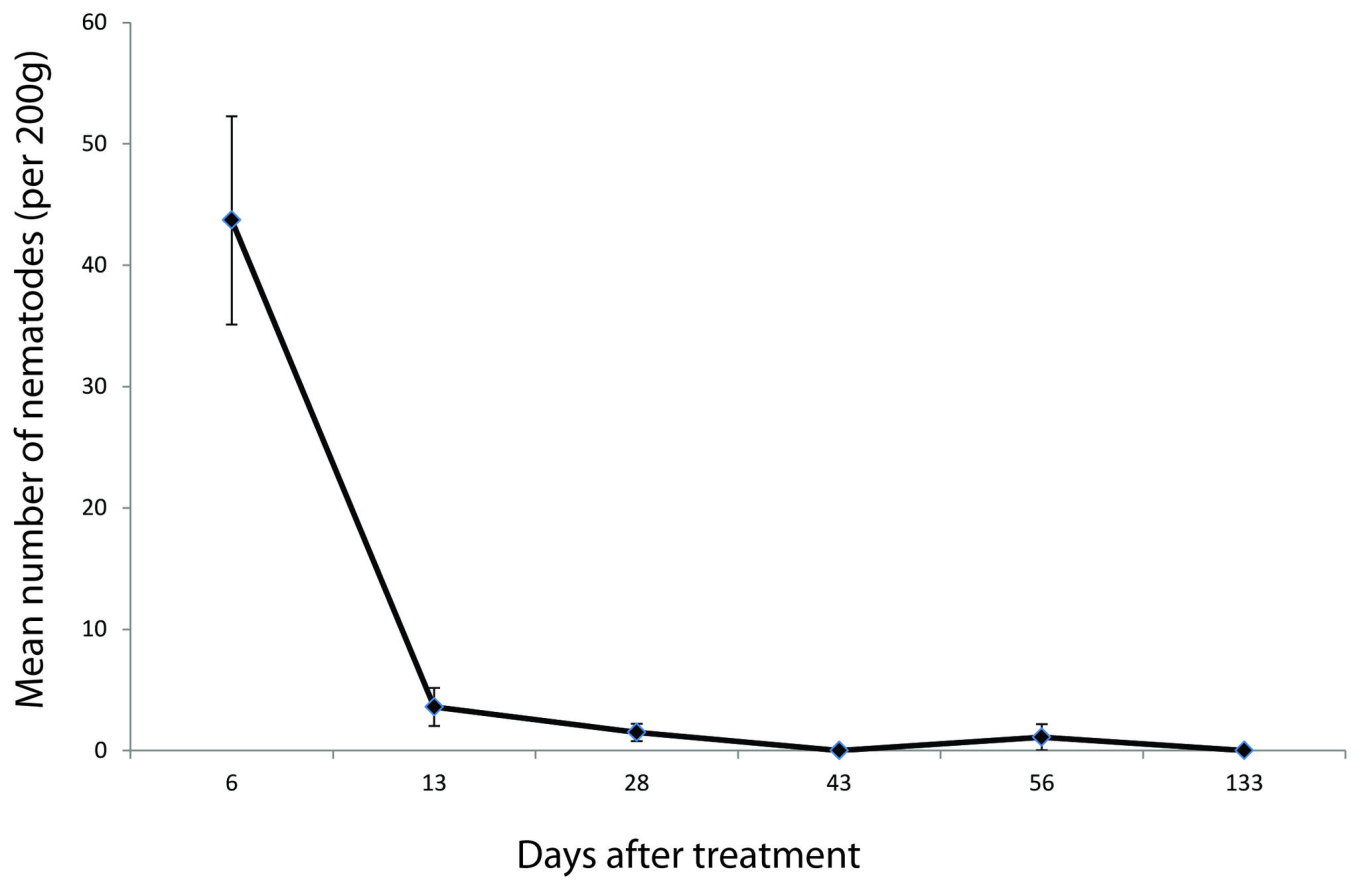
Persistence of applied *Phasmarhabditis hermaphrodita* in soil over time. Mean numbers of nematodes detected (real-time PCR) per 200g (SE error bars). N=5 (nematode treated plots).

There were no *P. hermaphrodita* detected in the soil samples taken before nematode application. A significant number of *P. hermaphrodita* was detected in soil samples from the treated plots directly after nematode application (Figure 3). However, the number of nematodes declined sharply to a low level in the first two weeks after treatment and none were detected after four months. Soil moisture was highest in May and August with an average of 61.3% (1.5 s.e.) and a range of 58 - 65%. Moisture was lowest in June with an average of 51.5% (5.5 s.e.) and a range of 46-57%. The density of *D. reticulatum*, *A. distinctus* and *A. silvaticus* was negatively related to the density of nematodes in the soil in early May, late May and June, respectively (Table 6). 

**Table 6:** Relationships between beetles (*Carabus nemoralis*), slugs and nematodes using generalised linear mixed-effect models (GLMMs).

	**Explanatory variables**	***Arion vulgaris***	***Arion silvaticus***	***Arion distinctus***	***Deroceras reticulatum***	**Beetle foregut mass**
**April**	Beetle activity-density	**0.5323**	NS	NS	**-0.5673**	-
	*A. vulgaris* positive beetles	**0.5864**	NS	NS	NS	**1.2552**
	*A. distinctus* positive beetles	NS	NS	NS	NS	**0.7562**
	*A. silvaticus* positive beetles	NS	**-0.2163**	NS	NS	NS
	*D. reticulatum* positive beetles	NS	NS	**1.1049**	NS	NS
	*A. vulgaris* density	-	NS	NS	**0.0885**	NS
	*A. silvaticus* density	NS	-	NS	NS	NS
	*A. distinctus* density	NS	NS	-	NS	NS
	*D. reticulatum* density	**0.1353**	NS	NS	-	NS
**Early May**	Beetle activity-density	NS	NS	NS	NS	-
	*A. vulgaris* positive beetles	**-3.0701**	NS	NS	NS	**0.2049**
	*A. distinctus* positive beetles	NS	**2.4331**	-	NS	NS
	*A. silvaticus* positive beetles	NS	NS	NS	**0.1254**	**0.4850**
	*D. reticulatum* positive beetles	NS	NS	NS	NS	**0.5045**
	*A. vulgaris* density	-	NS	NS	NS	**0.0267**
	*A. silvaticus* density	NS	-	NS	NS	**0.0149**
	*A. distinctus* density	**3.9952**	NS	-	NS	NS
	*D. reticulatum* density	NS	**0.1281**	NS	-	NS
	Nematode treatment	NS	NS	**-6.8832**	NS	**-1.0587**
	Nematode density in the soil	NS	NS	NS	**-0.0359**	**-0.0052**
	Infected slugs	NS	NS	**13.5182**	NS	**3.0754**
**Late May**	Beetle activity-density	NS	NS	NS	NS	-
	*A. vulgaris* positive beetles	NS	NS	NS	NS	NS
	*A. distinctus* positive beetles	NS	NS	-	NS	-
	*A. silvaticus* positive beetles	NS	**2.3318**	**-**	-	NS
	*D. reticulatum* positive beetles	NS	NS	NS	NS	NS
	*A. vulgaris* density	-	NS	NS	NS	NS
	*A. silvaticus* density	NS	-	NS	NS	NS
	*A. distinctus* density	NS	NS	-	NS	NS
	*D. reticulatum* density	**0.3778**	NS	NS	-	NS
	Nematode treatment	NS	NS	NS	**0.2693**	NS
	Nematode density in the soil	NS	NS	**-0.2682**	NS	NS
	Infected slugs	NS	NS	NS	**-0.7829**	NS
**June**	Beetle activity-density	NS	NS	NS	NS	-
	*A. vulgaris* positive beetles	NS	NS	NS	**3.7514**	**-8.0920**
	*A. distinctus* positive beetles	NS	NS	-	NS	NS
	*A. silvaticus* positive beetles	NS	NS	NS	NS	**2.2025**
	*D. reticulatum* positive beetles	NS	**-0.4464**	NS	NS	NS
	*A. vulgaris* density	-	NS	NS	NS	NS
	*A. silvaticus* density	NS	-	NS	**0.1120**	NS
	*A. distinctus* density	NS	**0.0720**	-	NS	NS
	*D. reticulatum* density	NS	**0.0510**	NS	-	**0.1427**
	Nematode treatment	NS	**-1.4229**	NS	NS	NS
	Nematode density in the soil	NS	**-0.1719**	NS	NS	NS
	Infected slugs	NS	**3.4932**	NS	NS	NS

Significant relationships are written in bold. “NS” = not significant.

### Predation and intraguild predation in the field

**Figure 4:**
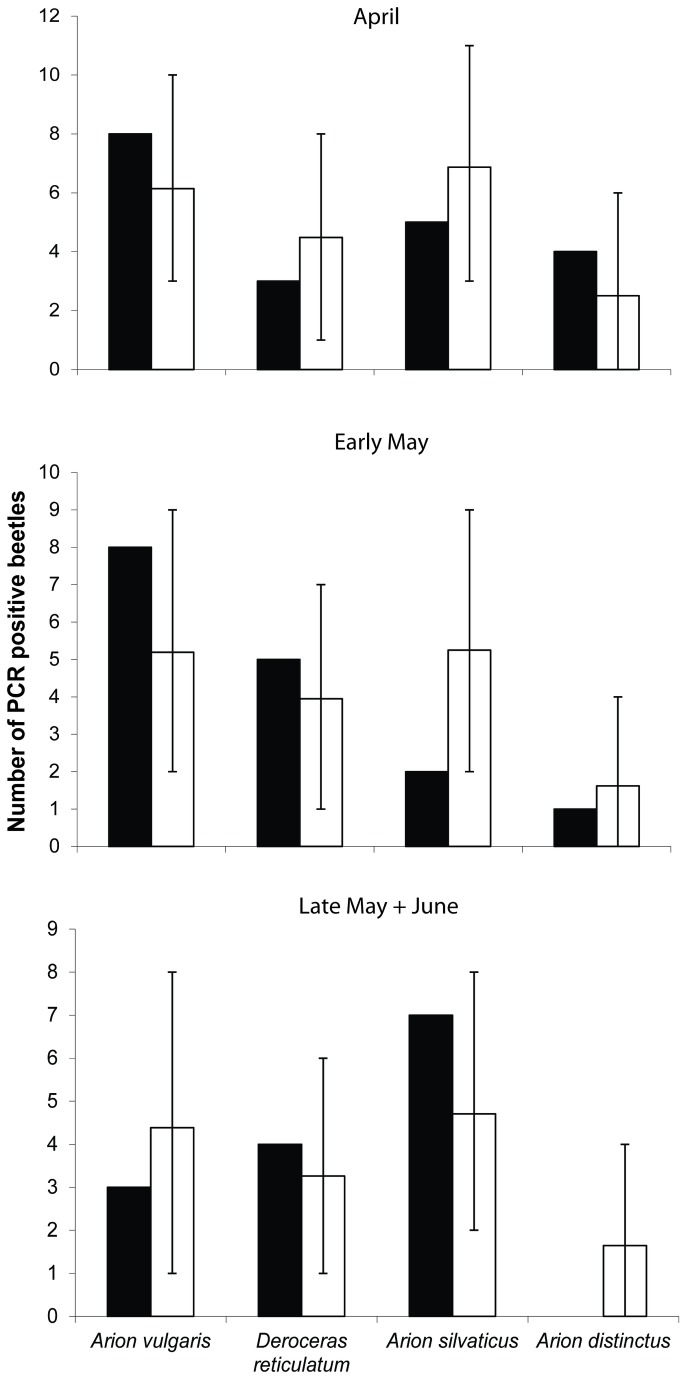
Comparison of observed numbers of *Carabus nemoralis* testing positive for slugs (black bars) with the expected consumption rates (open bars) based on a Monte Carlo model. Vertical bars indicate range of values enclosing the 95% confidence intervals.

We found no evidence for prey choice during the season and slug consumption mainly reflected the densities of the different slugs (Figure 4). The highest predation levels were on *A. vulgaris* at the beginning of the season, when more than 20% of *C. nemoralis* tested positive for *A. vulgaris* (Table 4) and when predation on other slug species was considerably lower. However, the number of beetles that tested positive for slug DNA was considerably lower in late May when mean mass of *A. vulgaris* reached more than one gram. This observation is also reflected in the comparison of observed vs. simulated PCR positives for late May/June (Figure 4). A higher number of slug-positive females relative to males was found (24 ♀ versus 15 ♂), but the difference was not quite significant (χ = 3.207, p = 0.073). The intraguild predation on nematode-infected slugs was low. Only three specimens of *C. nemoralis* were positive for nematode DNA and none of the P. *niger* or *P. melanarius* were positive for nematode-DNA. Two out of three nematode-positive *C. nemoralis* were also positive for *D. reticulatum* suggesting that these beetles had consumed slugs of the species which was most frequently infected by the nematodes. Furthermore, nine specimens of *C. nemoralis* were positive for several slug species, of which seven specimens were positive for two slug species while two specimens were positive for three slug species simultaneously. 

A positive relationship was found between foregut mass of beetles and the number of beetles being positive for *A. vulgaris* in April and early May, while this relationship was positive for *A. silvaticus* in early May and June (Table 6). The same relationship was also found for beetles being positive for *A. distinctus* DNA in April and *D. reticulatum* in early May. The foregut mass of beetles was also positively related to the density of *A. vulgaris* and *A. silvaticus* in early May. The activity-density of beetles, however, was only positively related to density of *A. vulgaris*, while being negatively related to the density of *D. reticulatum* in April. Furthermore, foregut mass of beetles was also only positively related to density of *A. vulgaris*. 

## Discussion

The aims of this study were to investigate predation, both temporally and spatially, on the invasive slug *A. vulgaris* and other slugs by carabid beetles. We also wanted to study effects of applying nematodes as biological control agents and investigate potential intraguild predation by the beetles on nematode-infected slugs.

The primers for slugs and nematodes proved to be species-specific and did not cross-amplify with non-target taxa. The detection period of nematode-DNA in beetle foreguts was rather short compared to slug-DNA, but sensitive even to low-level infection. The most abundant slugs in our field were *D. reticulatum*, *A. silvaticus*, *A. distinctus* and *A. vulgaris*, of which *D. reticulatum* was the most heavily infected by the nematodes. Predation by carabid beetles was most significant on *A. vulgaris*, which was also the most abundant slug species. Intraguild predation on the nematodes was low.

The effect of Nemaslug (*P. hermaphrodita*) did not last long. Furthermore, the application of nematodes had only a moderate effect on *A. vulgaris*, which has also been found in other field experiments [28, Haukeland et al. unpublished data]. However, there are reports of the successful use of *P. hermaphrodita* when applied against *D. reticulatum*, particularly in vegetable crops [28–30,54]. This also seemed to be the case in the present study indicated by high numbers of infected slugs and a negative relationship between nematodes in the soil and density of *D. reticulatum* on the first sampling date after the application of Nemaslug. 

Both pest slugs, including *A. distinctus*, and the non-pest slug *A. silvaticus* were negatively affected by nematode application, as indicated by high numbers of infected slugs. Furthermore, we found negative relationships between these *Arion* species and nematodes (Table 6). 

This is the first time a study of the persistence of released *P. hermaphrodita* in soil has been reported. The real-time PCR method [34] worked well and we were able to detect low densities of nematodes extracted from soil samples. We believe this method is suitable for further studies on populations of *P. hermaphrodita* in the soil environment. The results showed that the numbers of *P. hermaphrodita* recovered from soil samples declined sharply about two weeks after application. Similar results have been reported in field studies with other commercially applied nematodes (entomopathogenic nematodes, dauer larvae that infect insect larvae) [18]. Commercially cultured dauer larvae are prone to adverse effects from abiotic factors such as water logging, desiccation or direct sunlight soon after application. They may also face a variety of diseases and predators [39] in the soil environment. It is likely that large numbers of the applied nematodes succumbed to such mortality factors after application to the soil surface. Those that survived may not easily be detected over time in soil possibly due to the lifecycle options of these slug-parasitic nematodes [26]. Although not well studied there have been three distinct life cycles described: saprobic, necromenic and parasitic. The first has only been observed in the laboratory where *P. hermaphrodita* is able to grow on homogenized slugs as well as slug faeces [32] reproducing on a wide range of bacteria [27], thus the nematodes may persist in the environment without living slug hosts. The necromenic life cycle [33] is often observed in *Arion* species and some large *Limax* species, the nematodes (dauer larvae) can enter the slug and survive there without further development until the slug dies. The dauer larvae then recover and reproduce on the slug cadaver. The apparent non-susceptibility (no mortality) of many *Arion* species to *P. hermaphrodita* indicates that they may induce this life cycle [26, Haukeland unpublished data]. The parasitic life-cycle has been relatively well studied by Wilson et al. [31] and Tan and Grewal [32] in *D*. *reticulatum* where the slug usually dies one to four weeks after exposure to *P. hermaphrodita*. In this study we observed the parasitic life-cycle in *D. reticulatum* shortly after nematode application (12 days). The necromenic life-cycle was recorded at a low level in *A. vulgaris*, and at a higher level with *A. silvaticus*, we can only speculate on the reasons for this but it appears that *A. silvaticus* encountered nematodes more often than *A. vulgaris* and is possibly susceptible to fewer nematodes. The low level of nematodes detected after around eight weeks may indicate the presence of a new generation of dauer juveniles from *D. reticulatum* slug cadavers. However, improved methods using Nemaslug as a biological control agent of *A. vulgaris* are needed since the effects are clearly limited. Currently the use of *P. hermaphrodita* in slug baits such as animal feed pellets or bran are being tested, results so far indicate that this approach may be successful since slugs are attracted to and feed on such baits, later becoming infected with the nematodes [Haukeland, S. unpublished data]. 

Predation on slugs by *C. nemoralis* seems to be associated with higher densities of slugs both based on prey-DNA in gut analyses and density relationships, suggesting that these beetles are opportunistic slug-feeding carabids. These findings are in accordance with previous field studies on *C. nemoralis* that used PCR to analyze beetles [21], and suggest that predation on slugs is mainly density related. Little evidence of prey choice has also been found when examining carabid beetles in regard to predation on different earthworm species [46]. However, predation on *A. vulgaris* decreased significantly in late May when *A. vulgaris* reached a size of more than ~1g. This was supported by size-choice experiments and suggests a preference by *C. nemoralis* for slugs smaller than one gram [20]. The same pattern was also found by Paill [14,55] using isoelectric focusing [56], where predation on *A. vulgaris* by both adult and larvae of *Carabus violaceus*, as well as adult *P. melanarius*, was lowest when slugs were at their largest.

Significant predation on *A. vulgaris* was clearly shown by the number of beetles testing positive for *A. vulgaris* DNA. However, it was also indicated by positive relationships between foregut mass of beetles and slug-numbers, especially *A. vulgaris*. We also found positive relationships between slug-positive beetles and slug density, which is in accordance with Symondson et al. [57] who found that the proportion of slugs in beetles` diet were positively associated with slug density in the soil. These relationships varied during the season and were not as profound as found by Bohan et al. [5], where *P. melanarius* were aggregating to patches of slugs followed by a significant decrease in slug numbers. Digweed [58] showed that female *C. nemoralis* are able to orientate towards *D. reticulatum* and earthworms by following trails of mucus, while males only responded to earthworms. Furthermore, *C. nemoralis* have been shown to increase turning rate in mucus patches of *D. reticulatum* (klinotaxis) and even loop back and re-enter a patch after leaving it [59]. Slugs try to avoid areas containing chemical cues from predatory carabid beetles that include molluscs in their diets [60,61], which suggests that the interaction between these carabids and slugs are dynamic in the sense that carabids seek out slug patches while the slugs try to avoid the beetles. However, we found a random distribution of both beetles and *A. vulgaris* in our study in a field where the total prey diversity was probably high and alternative prey abundant. The lack of significant spatial aggregation may also have been a function of the scale of our sampled area, which may have been too small (ca 250m^2^). Previous studies have shown that carabids occur in clusters of at least one hectare in size [62], while smaller trapping scales may suggest uniform distributions [24]. Larger species of carabids, such as *Carabus* spp., are highly mobile and likely to forage over larger areas compared to smaller species [24]. 

Tritrophic interactions between beetles, slugs and nematodes seemed to be occurring. For example, we found a positive relationship between beetle foregut mass and infected slugs on the first sampling date after the nematode treatment when nematode densities were highest. This might suggest that the beetles were moving to patches where there were dead and debilitated slugs. However, only three beetles out of the total of 130 tested positive for nematode-DNA, suggesting that infected slugs may have been avoided or at least not preferred. The detection period of nematode-DNA was much shorter than for the slugs in our feeding experiment, possibly due to low infection levels in *A. vulgaris*. Infection levels of *A. vulgaris* were also low in the field, thus intraguild predation was probably underestimated. Previous studies [39], however, found a median detection time of 15.4 h after a 216 h infection period when using *D. reticulatum* as host, and also found that increasing infection levels in *D. reticulatum* led to longer detection time of *P. hermaphrodita*, while the detection time for *D. reticulatum* DNA decreased (from 16.1 h after 24 h of infection to 8.9 h after 216 h of infection). *Deroceras reticulatum* in our field were heavily infected, thus the detection time of nematode-DNA was probably longer than shown in our feeding experiment using *A. vulgaris*. Hence, potential intraguild predation on infected *D. reticulatum* should be as detectable as slug-DNA. A more likely explanation for the low detection of intraguild predation is the non-availability of highly infected slugs to beetles. Glen & Wilson [63] found that *D. reticulatum* undergoes torpor beneath the soil after being infected by *P. hermaphrodita*, thus being encountered less often by beetles. Furthermore, Foltan and Puza [38] found that *P. hermaphrodita* deter *P. melanarius* from feeding on slug infected cadavers, which is partly in accordance with [39] who found that these beetles prefer uninfected carrion over infected carrion in the later stages of infection (> 72h). However, beetles may not be able to distinguish between nematode infected and uninfected slugs during the first stage of infection, when the slugs are still alive and only contain a few nematodes. This may especially relate to *Arion* slugs, since *P. hermaphrodita* often persist in *Arion* slugs without killing their host according to our results. The nematodes are therefore unlikely to be visually apparent to the beetles, although there might be chemical cues or changes in slug activity of such infected slugs. However, if beetles were feeding significantly on live infected slugs the number of nematode-positive beetles should have been higher. 

Our results suggest that *C. nemoralis* may have the potential to have a significant impact on *A. vulgaris* in spring, and pesticide use as well as cultivation disturbance should be as limited as possible during this time of year to reduce negative effects on beetle numbers. In addition, semi-natural habitats surrounding crop fields are important to enhance carabid assemblages including woodland-edge species such as *C. nemoralis* and *P. niger* [64]. Our findings also suggest that Nemaslug can be applied for biological control in spring when slug-feeding beetles such as *C. nemoralis* are active, since intraguild predation seems to be low. Further studies should be carried out on intraguild predation to support these findings involving semi-field experiments to test beetle preferences for different slugs in different infection stages. Nevertheless, the present study provides new insights into a prey-parasite-predator system under natural conditions with major implications for future work. 

## Supporting Information

File S1 **Feeding experiments to analyse DNA detection periods.**
(PDF)

File S2 **Extraction protocols, PCR conditions and cross-amplification tests.**
(DOC)
