# Supplementary material for: Spatiotemporal Analysis of Predation by Carabid Beetles (Carabidae) on Nematode Infected and Uninfected Slugs in the Field
Source: PLoS One. 2013 Dec 12;8(12):e82142. doi: 10.1371/journal.pone.0082142 (PMC3861370; doi:10.1371/journal.pone.0082142)
Supplement: File S1 — Feeding experiments to analyse DNA detection periods. (PDF) [file pone.0082142.s001.pdf]

Feeding experiments to analyse DNA detection periods

Slugs and beetles were collected around the perimeter of the same field as used for the field experiment mentioned above. All specimens were kept at 14 °C in a climate chamber (RUMED® Rubarth Apparate GmbH) simulating the light conditions from the field following a light regime of 50% of maximum light from 6:00 to 8:00, 100% from 8:00 to 20:00, and 50% from 20:00 to 22:00 followed by darkness from 22:00 to 6:00, both prior to and during the feeding experiment. Two similar sized slugs ranging in size from 0.2 g to 0.5 g were placed in Petri dishes 78 mm in diameter containing a moist filter paper. The sides of the dishes were coated with Fluon® to prevent the slugs climbing the sides and thus avoiding the nematodes. Slugs of these sizes were chosen since they were the most abundant in the field. Beetles were fed with earthworms (*Lumbricus rubellus*) to equalise their state of nutrition and conditioning, and then starved for 10 days prior to use in feeding experiments.

In the parasite-host experiment we prepared the nematodes and applied them following the manufacturers recommended rate at 30 dauer larvae per cm^2 in 900 μL tap water. The slugs (*A. vulgaris*) were exposed to nematodes for a week. One of the slugs from each dish was frozen at -80 °C and later assessed for the infection level. The remaining slugs were fed to *C. nemoralis*, one slug per beetle. After a 2 h feeding period, the beetles were put back in their plastic boxes with moist vegetation. Beetles were killed inside the containers at -20 °C in batches of three males and three females at the following time-points; 2, 12, 24 and 48 h after the mid-point of the feeding period. Twelve unfed beetles were frozen as controls. All beetles were stored at -80 °C prior to DNA extraction.

In the detection period experiments using *A. silvaticus* and *A. distinctus* we also used a 2 h feeding period. The beetles were then killed and stored using the same conditions as above, but using batches of four males and four females killed at the following time-points; 2, 10, 20, 40 and 80 h after the mid-point of the feeding period. Eight unfed beetles were frozen as controls.
