# Supplementary material for: Spatiotemporal Analysis of Predation by Carabid Beetles (Carabidae) on Nematode Infected and Uninfected Slugs in the Field
Source: PLoS One. 2013 Dec 12;8(12):e82142. doi: 10.1371/journal.pone.0082142 (PMC3861370; doi:10.1371/journal.pone.0082142)
Supplement: File S2 — Extraction protocols, PCR conditions and cross-amplification tests. (DOC) [file pone.0082142.s002.doc]

**S2 Extraction protocols, PCR conditions and cross-amplification tests**

*DNA extractions*

DNA was extracted from slugs and beetles using the Qiagen DNeasy Blood & Tissue kit and stored in elution buffer at -80 °C. General invertebrate primers amplifying part of the mitochondrial COI gene [1] were used as controls, confirming extraction of amplifiable DNA. Any negatives were excluded from further analyses. All PCRs were repeated two or more times with different buffer solutions (GoTaq and Qiagen Multiplex) to confirm the final result. DNA from the nematodes extracted from soil was isolated using a soil DNA extraction kit (FastDNA SPIN kit, Q-BIOgene, Illkirch, France). The DNA was purified using Micro Bio Spin Chromatography columns (BioRad Laboratories Ltd., Hertfordshire, UK) filled with insoluble polyvinylpolypyrrolidone (PVPP).

*PCR evaluations and optimizations*

One of the multiplexes [2] included species-specific primers amplifying mitochondrial COI DNA from *Arion ater*, *A. vulgaris* and *A. rufus*. Each PCR was conducted in 12.5 μl, containing 6.25 μl GoTaq (Promega) or Qiagen Multiplex PCR Mastermix, 0.25 μl of each primer (10 µM), 0.125 µl BSA (10 mg/ml), 1.0 μl DNA and 3.875 μl dH_2_O. Cycling conditions of 94 °C for 15 min, followed by 30 cycles of 92 °C for 30 sec, 51 °C for 1 min and 72 °C for 50 sec, and a final cycle of 68 °C for 5 min were used when applying Qiagen Multiplex PCR Mastermix, while denaturing for 2 min, annealing for 30 sec, and extension of 70 °C were carried out when using the GoTaq PCR Mastermix. All PCR runs included positive (target prey) and negative controls. The latter consisted of PCR reagents with distilled water as substitute for DNA.

The other multiplex was designed by Harper *et al.* [3]. These mitochondrial 12S primers were developed by Dodd [4] and include an *Arion*-specific primer pair that amplifies different sized fragments for each *Arion* species, and species-specific primers for *D. reticulatum*. All PCRs involving these primers were conducted in 10 μL, containing 5 μL Qiagen Multiplex PCR Mastermix, 0.25 μL of each primer (10 µM), 0.1 µl BSA (10 mg/ml), 1.0 μL DNA and 2.9 μL dH_2_O. Cycling conditions were 95 °C for 15 min, followed by 40 cycles of 94 °C for 30 sec, 53 °C for 1.5 min and 72 °C for 1.5 min, and a final cycle of 72 °C for 10 min. All PCRs were carried out in a MJ Research PTC220 Peltier thermal cycler and PCR products were checked on 1-2% agarose gels.

The standard PCR for detection of the slug *A. silvaticus* in beetle foreguts was designed to amplify mitochondrial COI DNA (Table 1). The PCRs were performed in 10 μL, containing 5 μL Qiagen Multiplex PCR Mastermix, 0.5 μL of each primer (10 µM), 0.1 µl BSA (10 mg/ml), 1.0 μL DNA and 2.9 μL dH_2_O. Cycling conditions were 95 °C for 15 min, followed by 30 cycles of 92 °C for 30 sec, 54 °C for 30 sec and 70 °C for 50 sec, and a final cycle of 68 °C for 5 min.

The standard PCR for detection of the nematode *P. hermaphrodita* in beetle foreguts was designed by Read [5] and amplifies mitochondrial COI DNA. The PCRs were performed in 10 μL, containing 5 μL GoTaq (Promega) or Qiagen Multiplex PCR Mastermix, 0.5 μL of each primer (10 µM), 0.1 µl BSA (10 mg/ml), 1.0 μL DNA and 2.9 μL dH_2_O. Cycling conditions were 94 °C for 15 min, followed by 35 cycles of 94 °C for 30 sec, 62 °C for 30 sec and 72 °C for 1 min, and a final cycle of 72 °C for 10 min when using Qiagen Multiplex PCR Mastermix.

Real-time PCR for quantification of *P. hermaphrodita* nematodes extracted from soil samples was performed using primers and a FAM-labelled probe targeting 18S rRNA [6]. A standard curve made from known numbers of nematodes was used to convert *Ct* values (calculated automatically by the real-time PCR system) to number of nematodes in the samples [6]. A non-template control consisting of dH_2_O instead of DNA, and a positive control with template with known numbers of nematodes, were included in each PCR run. All reactions were performed in duplicate. Amplifications were performed using an ABI Prism 7900HT Fast Real-Time PCR system with standard block, combined with 7900HT Version 2.3 Sequence Detection Systems Software (Applied Biosystems).

*Cross-amplification tests on non-target organisms*

A total of 54 potential prey species from different taxa (16 gastropods, 3 oligochaeta, 10 arachnids, 1 isopod, 3 millipedes and 28 insects including 4 collembolans), as well as the three predators investigated in this study (*C. nemoralis*, *P. niger* and *P. melanarius*), were tested for PCR amplification using the standard PCR tests for *P. hermaphrodita* and *A. silvaticus*. The same samples as used in Hatteland *et al.* [2] were included in these non-target tests. Both multiplex reactions have been tested previously using the same samples [2]. In addition, Read [5] tested the primer pair for the nematode using 100 additional non-target organisms. Species specificity of the real-time PCR assay has also been tested against a range of nematode parasites of invertebrates [6], since soil extracted nematodes may contain nematodes other than *P. hermaphrodita*.

1. Folmer O, Black M, Hoeh W, Lutz R & Vrijenhoek R (1994) DNA primers for amplification of mitochondrial cytochrome c oxidase subunit I from diverse metazoan invertebrates. Mol Mar Biol Biotechnol*,* 3, 294-299.
2. Hatteland B A, Symondson WOC, King RA, Skage M, Schander C et al. (2011) Molecular analysis of predation by carabid beetles (Carabidae) on the invasive Iberian slug *Arion lusitanicus*. Bull Ent Res 101: 675-686.
3. Harper GL, King RA, Dodd CS, Harwood JD, Glen DM et al. (2005) Rapid screening of invertebrate predators for multiple prey DNA targets. Mol Ecol 14: 819-827.
4. Dodd CS (2004) Development and optimisation of PCR-based techniques in predator gut content analysis [PhD thesis], Cardiff: Cardiff University.
5. Read D (2007) Molecular analysis of subterranean detrivore food webs [PhD thesis], Cardiff: Cardiff University.
6. MacMillan K, Blok V, Young I, Crawford J & Wilson MJ (2006) Quantification of the slug parasitic nematode *Phasmarhabditis hermaphrodita* from soil samples using real time qPCR. J Parasitol*,* 36, 1453-1461.
